# Supplementary material for: Interplay between T3SS effectors, ExoY activation, and cGMP signaling in Pseudomonas aeruginosa infection
Source: Nat Commun. 2025 Dec 2;17:69. doi: 10.1038/s41467-025-66674-z (PMC12770572; doi:10.1038/s41467-025-66674-z)
Supplement: Supplementary file 1 — Supplementary Information [file 41467_2025_66674_MOESM1_ESM.pdf]

Supplementary Information

Interplay between T3SS effectors, ExoY activation, and cGMP signaling in *Pseudomonas aeruginosa* infection

Vincent Deruelle[1,*], Gabrielle Dupuis[1,2], Dorothée Raoux-Barbot[1], Elysa Lim[1,3], Roberto Ponce-López[1], Magda Teixeira Nunes[4], Daniel Ladant[1], Louis Renault[4] and Undine Mechold[1]

[1]Institut Pasteur, Université Paris Cité, CNRS UMR3528, Biochemistry of Macromolecular Interactions Unit, Paris, France
[2]Centre de Recherche Saint-Antoine (CRSA), Sorbonne Université, INSERM UMR S 938, 5PMed : Pulmonary diseases, Pathogens, Physiopathology, Phenogenomics and Personalized Medecine, Paris, France
[3]Present address: School of Biomolecular and Biomedical Science and Michael Smurfit Graduate Business School, University College Dublin, Ireland
[4]Université Paris-Saclay, CEA, CNRS, Institute for Integrative Biology of the Cell (I2BC), Gif-sur-Yvette, France
* Correspondence: vincent.deruelle@pasteur.fr

Supplementary Figures 1–6, Supplementary Tables 1 and 2.

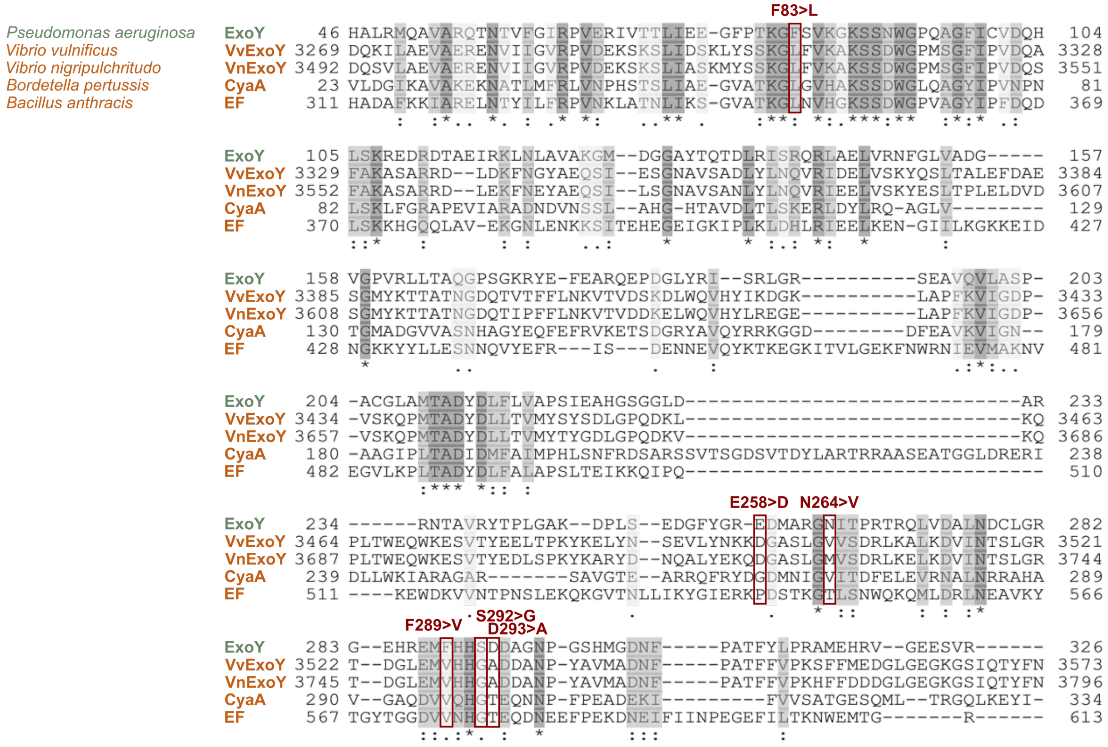

Supplementary Fig. 1: Alignment of cAMP-inducing toxins with ExoY effector from *Pseudomonas aeruginosa*.

Multiple sequence alignment of adenylate cyclase toxins from *Bordetella pertussis* and *Bacillus anthracis* (CyaA and EF respectively) and the ExoY-like modules from *Vibrio vulnificus* and *V. nigripulchritudo* (VvExoY and VnExoY respectively) with ExoY effector from *Pseudomonas aeruginosa* using the Clustal. Residues have been shaded to indicate different levels of conservation, with strictly conserved amino acids shown in dark gray and similar amino acids shown in lighter gray. A residue missing in one effector relative to the others is represented by "-". An asterisk "*" indicates that the residue is fully conserved among the effectors. Two dots ":" indicate a conserved substitution while one dot "." indicates semi-conserved substitutions. Numbers at the beginning and end of each sequence denote the amino acid positions. The four mutations selected in the nucleotide binding pocket of ExoY to change its substrate specificity are indicated in red.

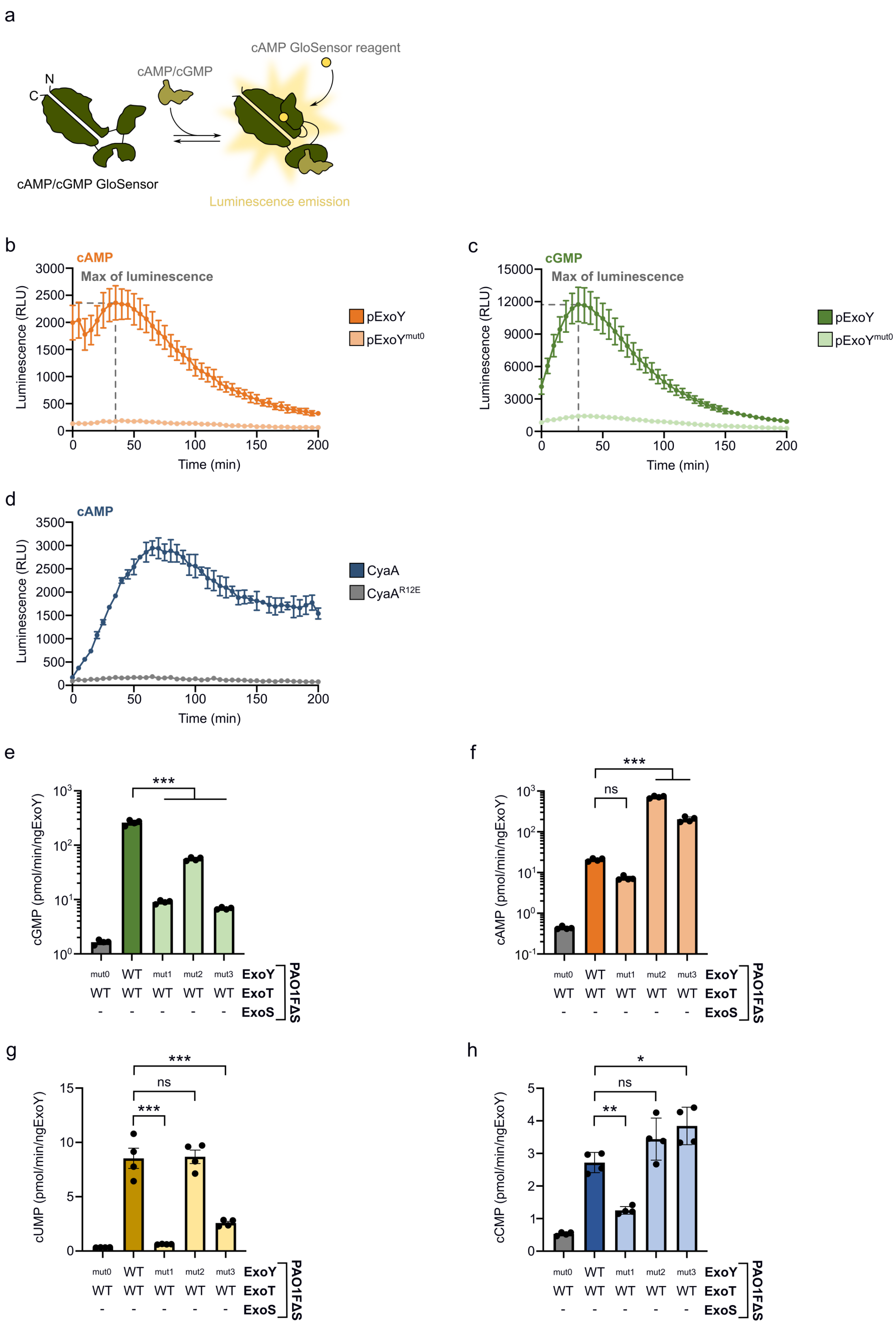

Supplementary Fig. 2: Luminescence emission from cAMP- and cGMP-reporter cell lines.
a. Representation of cAMP or cGMP Glosensor structure before and after interaction with second messengers (based on Promega website). Binding of the analyte (cAMP or cGMP)

induces conformational changes in the GloSensor, which emits luminescence following addition and binding of the Glosensor cAMP reagent. The magnitude of luminescence is proportional to analyte concentration. **b.** Increased luminescence emitted by stable clonal NCI-H292 cells expressing the cAMP- or **c.** cGMP Glosensor. Two days before acquisition, cells were co-transfected with plasmids encoding ExoY or catalytically inactive ExoY ($ExoY^{mut0}$) under control of the *TRE3G* promoter (P_{TRE3G}) and the transactivator required for activation. Prior to measurement, transfected cells were incubated with 1 µg/mL Doxycycline for 3h to trigger *exoY* expression. The cell medium was then replaced with fresh cell medium supplemented with 5% Glosensor cAMP reagent. Acquisitions were performed at 24°C every 5 minutes for 200 minutes with an integration time of 1000 ms. The dashed line indicates the maximum luminescence values as reported in Figure 1 F and G. **d.** Increased luminescence emitted by stable clonal cell line expressing the cAMP Glosensor after intoxication with 1 nM of CyaA WT or $CyaA^{R12E}$ proteins. Recombinant $CyaA^{R12E}$ protein does not translocate and was used as a negative control[2]. Cells were incubated for 1 h in CyaA activation medium supplemented with 5% Glosensor cAMP reagent prior to the addition of CyaA. Acquisitions were performed at 24°C every 3 minutes for 200 minutes with an integration time of 1000 ms. Error bars indicate SD. **e.** Analysis of guanylate cyclase (GC), **f.** adenylate cyclase (AC), **g.** uridylyl cyclase (UC), and **h.** cytidylyl cyclase activities in bacterial supernatants containing ExoY WT or one of the ExoY variants. '-' means that the effector is not expressed due to deletion of the corresponding gene. Data of biological replicates represented as mean ± SD, n = 4 (e, f, g, h). Statistical differences were established by one way ANOVA followed by Dunnett's multiple comparison test with the supernatant containing WT ExoY as a control group. Exact P values (e): PAO1FΔS(T,Y) vs. PAO1FΔS(T,Y^{mut1}) or PAO1FΔS(T,Y^{mut2}) or PAO1FΔS(T,Y^{mut3}) = < 0.001, (f): PAO1FΔS(T,Y) vs. PAO1FΔS(T,Y^{mut2}) or PAO1FΔS(T,Y^{mut3}) = < 0.001, (g): PAO1FΔS(T,Y) vs. PAO1FΔS(T,Y^{mut1}) or PAO1FΔS(T,Y^{mut3}) = < 0.001, (h): PAO1FΔS(T,Y) vs. PAO1FΔS(T,Y^{mut1}) = 0.002, PAO1FΔS(T,Y) vs. PAO1FΔS(T,Y^{mut3}) = 0.01. *p=0.033, **p=0.002, ***p<0.001, n.s., non-significant. Source data are provided as a Source Data file.

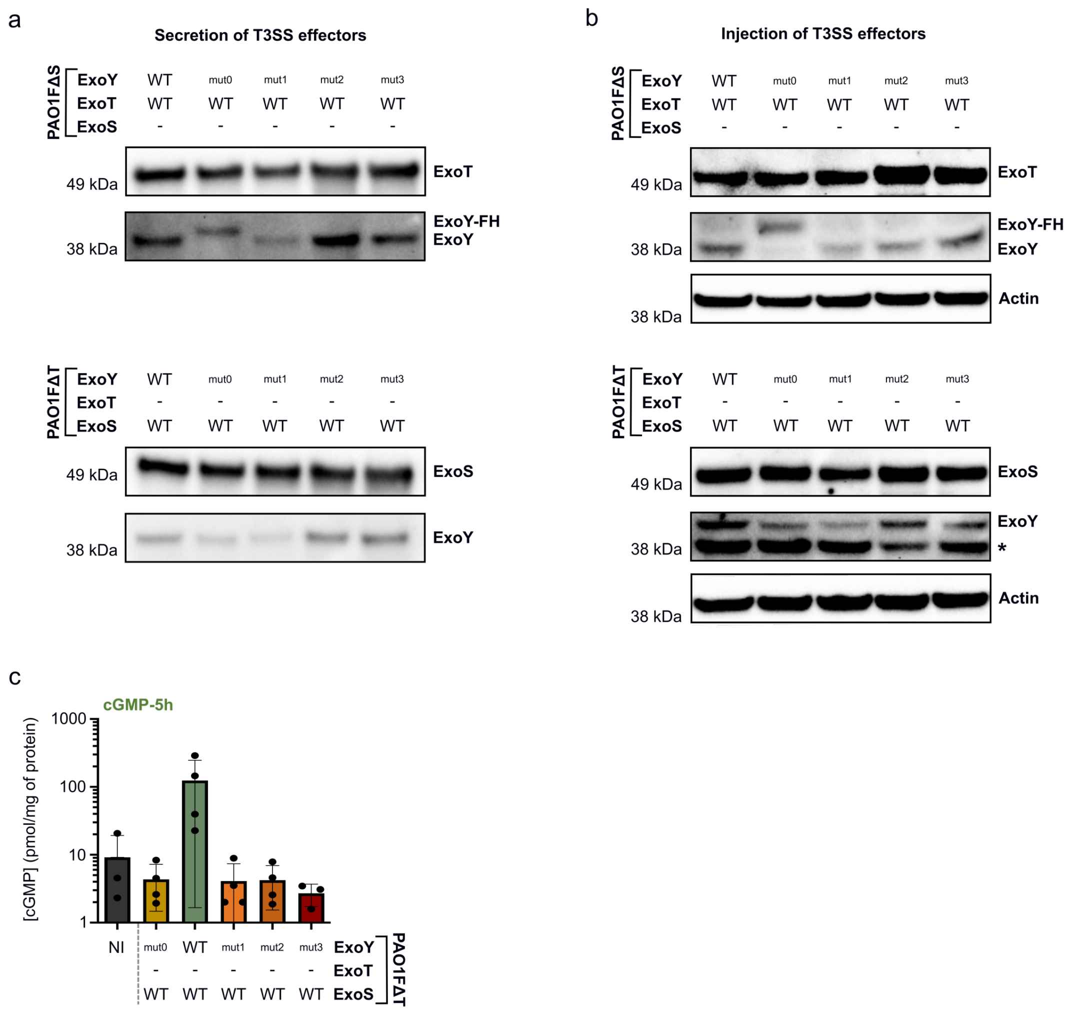

Supplementary Fig. 3: Analysis of secretion and injection of T3SS effectors and cGMP production by PAO1FΔS(T,Y) or PAO1FΔT(S,Y) strains.

a. Western blot (WB) of secreted T3SS effectors in bacterial culture supernatants. Overnight cultures of PAO1FΔS(T,Y) and PAO1FΔT(S,Y) strains were diluted to 0.05 and grown for 3 h in LB supplemented with 5 mM EGTA and 20 mM $MgCl_2$ to secrete ExoT/ExoS and the different ExoY variants. The $ExoY^{mut0}$ variant in the PAO1FΔS(T,Y) strain has a higher molecular weight because it contains a Flag-His tag at its C-terminus. **b.** WB showing T3SS effectors injected into NCI-H292 cell line after infection for 5h at an MOI of 20 with PAO1FΔS(T,Y) and PAO1FΔT(S,Y) strains. The $ExoY^{mut0}$ variant in the PAO1FΔS(T,Y) strain has a higher molecular weight because it contains a Flag-His tag at its C-terminus. T3SS effectors were revealed with anti ExoS, ExoT or ExoY antibodies. β-actin was used as loading control. (*) represents a non-specific band corresponding to a cellular protein that reacts with our ExoY antibody, as this band is also visible in the uninfected condition. The WBs presented correspond to one experiment.**c.** Quantification of cGMP production in NCI-H292 cells by ELISA after 5h of infection at MOI 20 with PAO1FΔT(S,Y) strains co-injecting ExoS with an ExoY variant. '-' means that the effector is not expressed due to deletion of the corresponding gene. NI means 'not infected'. Data of biological replicates represented as mean ± SD, n = 3. Source data are provided as a Source Data file. Uncropped scans of the blots are provided at the end of the Supplementary Information file.

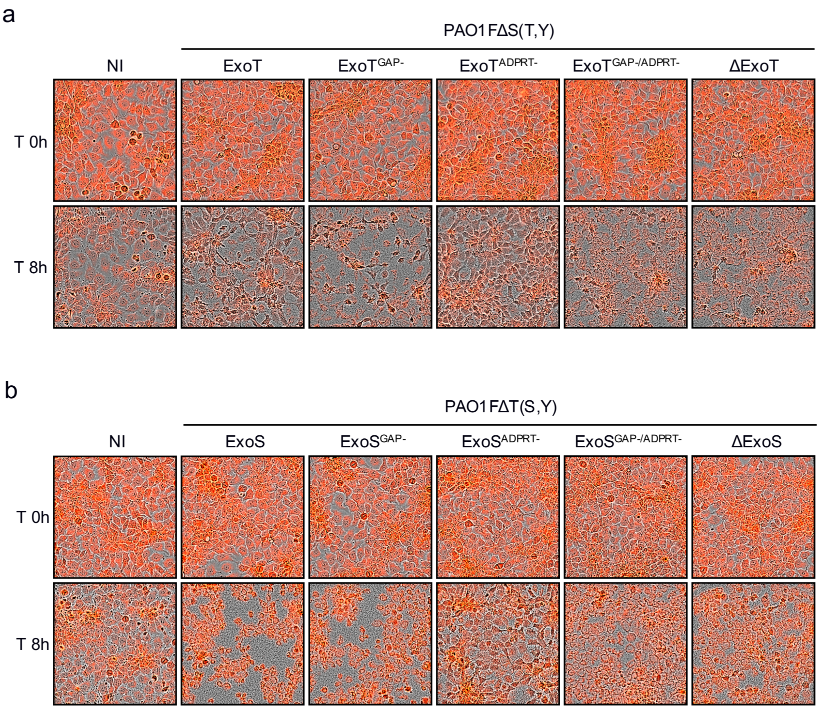

Supplementary Fig. 4: Visualization of cell retraction induced by ExoT, ExoS or their derivatives.

a. Images showing cell rounding induced by ExoT activities. Cells were incubated with 1 µM Cytotrace to label the cell cytoplasm and infected at MOI 50 for 8h with PAO1FΔS(T,Y) strains injecting ExoT toxin or its derivatives. Representative images show merged acquisition of phase contrast and Cytotrace fluorescence (red) at the beginning (T 0h) and after 8 hours of infection (T 8h). **b.** Representative images of cell rounding induced by ExoS activities. Similar to **a.** but cells were infected at MOI 20 for 8h with PAO1FΔT(S,Y) strains injected ExoS toxin or its derivatives. NI means 'not infected'.

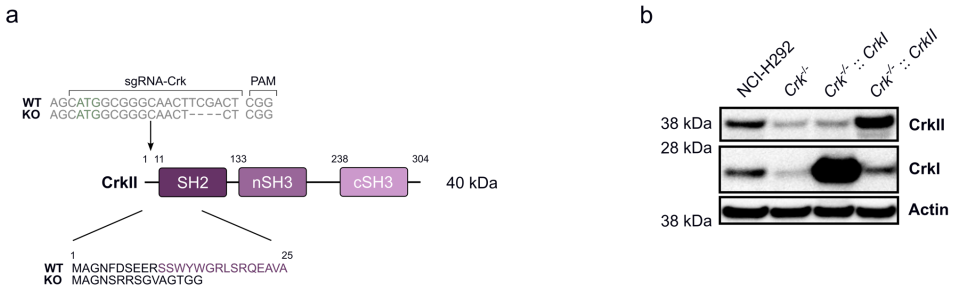

Supplementary Fig. 5: Analysis of phospho-CrkII and Crk family proteins expression in native and Crk-deficient NCI-H292 cells.

a. Alignments of native *crkII* sequence (WT) and mutant allele (KO) at the level of DNA and protein sequence showing four missing nucleotides in the mutant allele. The start codon is represented in green. The PAM sequence and the sequence of the sgRNA used for inactivation are shown. The mutant allele results in the expression of a truncated protein due to the introduced frame shift. The result is expected to be the same for CrkI as the deletion is at the beginning of the *crk* gene. **b.** WB of NCI-H292 and monoclonal Crk-/- NCI-H292 complemented or not with plasmids expressing CrkI or CrkII. The Crk proteins were revealed with the mouse recombinant monoclonal Crk antibody, targeting both proteins. β-actin was used as loading control. The WB presented correspond to one experiment. Source data are provided as a Source Data file. Uncropped scans of the blots are provided at the end of the Supplementary Information file.

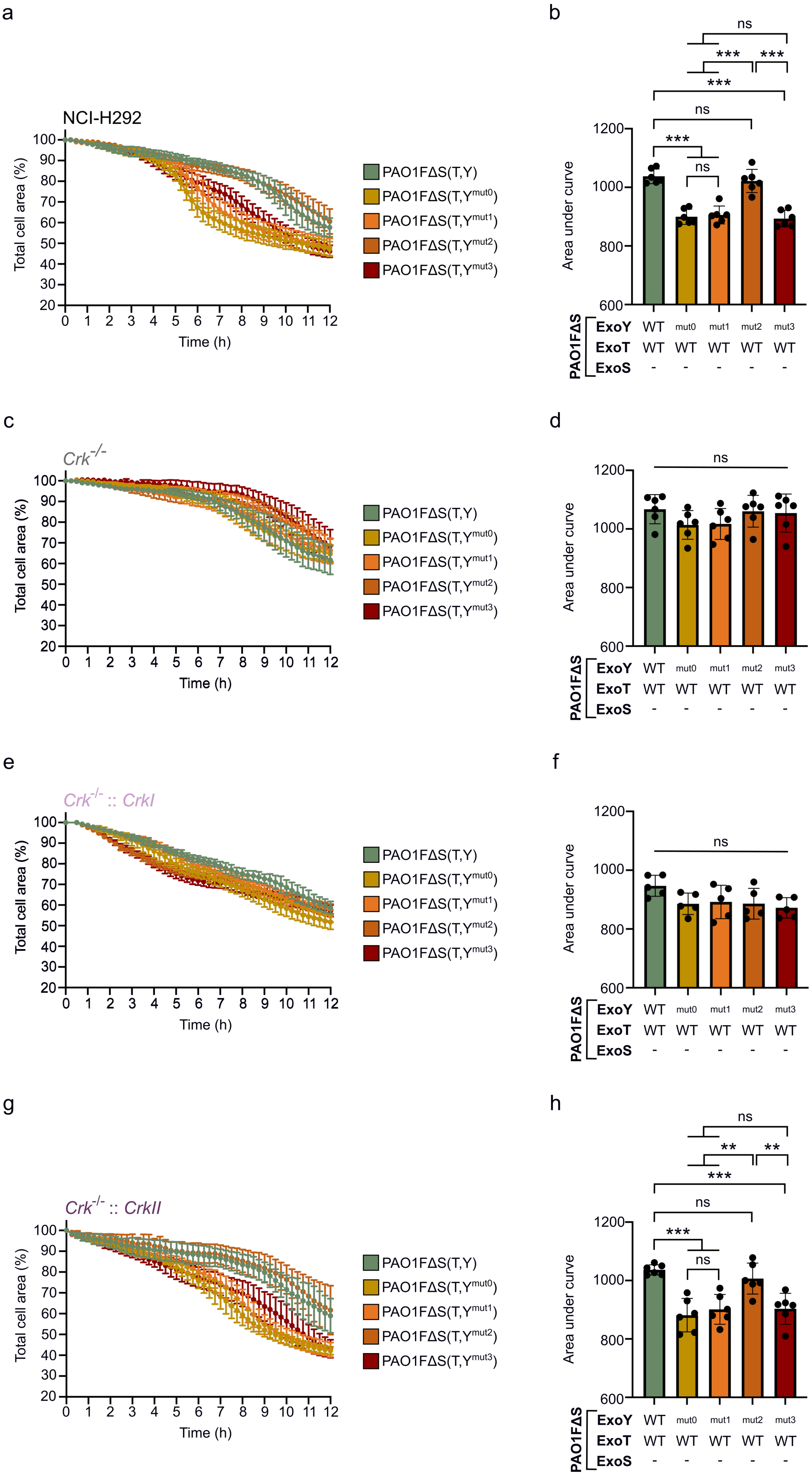

Supplementary Fig. 6: ExoT-induced cell retraction in NCI-H292 WT or Crk-deficient cells, complemented or not with CrkI or CrkII protein.

a. ExoT-induced cell retraction over time in NCI-H292 cells, **c.** monoclonal Crk-deficient cells, **e.** monoclonal Crk-deficient cells complemented with CrkI, and **g.** monoclonal Crk-deficient

cells complemented with CrkII. Cytotrace was used to label the cell cytoplasm and cells were infected at MOI of 20 with PAO1FΔS(T,Y) strains co-injecting ExoT with an ExoY variant. Intoxication was recorded every 15 min by time-lapse microscopy. Results are represented as the mean percentage (±SD) of total cell area. Panels **b.**, **d.**, **f.**, and **h.** correspond to quantifications of the area under the curve (AUC) for each infection curve. The lower the AUC, the more cytotoxic the strain. Each dot represents a separate cell retraction assay (biological replicate). '-' means that the effector is not expressed due to deletion of the corresponding gene. Data of biological replicates represented as mean ± SD, n = 6 (b, d, h), n = 5 (f). Statistical differences were established by one-way ANOVA followed by Tukey's multiple comparison test. Exact P values (b): PAO1FΔS(T,Y) vs. PAO1FΔS(T,Y^{mut0}) or PAO1FΔS(T,Y^{mut1}) or PAO1FΔS(T,Y^{mut3}) = < 0.001, PAO1FΔS(T,Y^{mut0}) or PAO1FΔS(T,Y^{mut1}) vs. PAO1FΔS(T,Y^{mut2}) = < 0.001, PAO1FΔS(T,Y^{mut2}) vs. PAO1FΔS(T,Y^{mut3}) = < 0.001, (h): PAO1FΔS(T,Y) vs. PAO1FΔS(T,Y^{mut0}) or PAO1FΔS(T,Y^{mut1}) or PAO1FΔS(T,Y^{mut3}) = < 0.001, PAO1FΔS(T,Y^{mut0}) vs. PAO1FΔS(T,Y^{mut2}) = 0.001, PAO1FΔS(T,Y^{mut1}) vs. PAO1FΔS(T,Y^{mut2}) = 0.007, PAO1FΔS(T,Y^{mut2}) vs. PAO1FΔS(T,Y^{mut3}) = 0.009. *p=0.033, **p=0.002, ***p<0.001, n.s., non-significant. Source data are provided as a Source Data file.

Supplementary Table 1: Modified *CrkI* and *CrkII* genes synthesized by Twist

**Names**	**Sequence (5'→ 3')***
Modified *CrkI*	TCTAGAGCCATGGCGGGAAATTTTGATTCTGAGGAGCGGAGTAGCTGGTACTG<br>GGGGAGGTTGAGTCGGCAGGAGGCGGTGGCGCTGCTGCAGGGCCAGCGGCA<br>CGGGGTGTTCCTGGTGCGGGACTCGAGCACCAGCCCCGGGGACTATGTGCTCA<br>GCGTCTCAGAGAACTCGCGCGTCTCCCACTACATCATCAACAGCAGCGGCCC**TA**<br>G**A**CC**T**CCGGTGCCACCGTCGCC**T**GCCCAGCCTCC**T**CC**T**GGGGTGAGCCCCTCCA<br>GACTCCGAATAGGAGATCAAGAGTTTGATTCATTGCCTGCTTTACTGGAATTCTA<br>CAAAATACACTATTTGGACACTACAACGTTGATAGAACCAGTTTCCAGATCCAG<br>GCAGGGTAGTGGAGTGATTCTCAGGCAGGAGGAGGCGGAGTATGTGCGAGCC<br>CTCTTTGACTTTAATGGGAATGATGAGGAAGATCTTCCCTTTAAGAAAGGAGAC<br>ATCTTGAGAATCCGGGACAAGCCTGAAGAGCAGTGGTGGAATGCGGAGGACA<br>GCGAAGGCAAGAGAGGGATGATTCCAGTCCCTTACGTCGAGAAGTATAGACCT<br>GCCTCCGCCTCAGTATCGGCTCTGATTGGAGGTCGGTGAGGATCC
Modified *CrkII*	TCTAGAGCCATGGCGGGAAATTTTGATTCTGAGGAGCGGAGTAGCTGGTACTG<br>GGGGAGGTTGAGTCGGCAGGAGGCGGTGGCGCTGCTGCAGGGCCAGCGGCA<br>CGGGGTGTTCCTGGTGCGGGACTCGAGCACCAGCCCCGGGGACTATGTGCTCA<br>GCGTCTCAGAGAACTCGCGCGTCTCCCACTACATCATCAACAGCAGCGGCCC**TA**<br>G**A**CC**T**CCGGTGCCACCGTCGCC**T**GCCCAGCCTCC**T**CC**T**GGGGTGAGCCCCTCCA<br>GACTCCGAATAGGAGATCAAGAGTTTGATTCATTGCCTGCTTTACTGGAATTCTA<br>CAAAATACACTATTTGGACACTACAACGTTGATAGAACCAGTTTCCAGATCCAG<br>GCAGGGTAGTGGAGTGATTCTCAGGCAGGAGGAGGCGGAGTATGTGCGAGCC<br>CTCTTTGACTTTAATGGGAATGATGAGGAAGATCTTCCCTTTAAGAAAGGAGAC<br>ATCTTGAGAATCCGGGACAAGCCTGAAGAGCAGTGGTGGAATGCGGAGGACA<br>GCGAAGGCAAGAGAGGGATGATTCCAGTCCCTTACGTCGAGAAGTATAGACCT<br>GCCTCCGCCTCAGTATCGGCTCTGATTGGAGGTAACCAGGAGGGTTCCCACCCA<br>CAGCCACTGGGTGGGCCGGAGCCTGGGCCCTATGCCCAACCCAGCGTCAACAC<br>TCCGCTCCCTAACCTCCAGAATGGGCCCATATATGCCAGGGTTATCCAGAAGCG<br>AGTCCCCAATGCCTACGACAAGACAGCCTTGGCTTTGGAGGTCGGTGAGCTGG<br>TAAAGGTTACGAAGATTAATGTGAGTGGTCAGTGGGAAGGGGAGTGTAATGG<br>CAAACGAGGTCACTTCCCATTCACACATGTCCGTCTGCTGGATCAACAGAATCCC<br>GATGAGGACTTCAGCTGAGGATCC

* Red letters represent mutations that prevent the Cas9 endonuclease from cleaving the gene. Bold letters represent silent mutations aimed at reducing %GC content. The yellow and blue letters correspond to the XbaI and BamHI restriction sites respectively.

Supplementary Table 2: Number of independent repetitions for each experiment

Figures	Number of independent experiments	Supplementary figures	Number of independent experiments
1B	3	2E	4
1C	3	2F	4
1D	3	2G	4
1E	3	2H	4
1F	4	3C	3
1G	3	6B	6
1H	4	6D	6
1I	6	6F	5
2B	3	6H	6
2D	5		
2F	4		
3B	3		
3C	4		
3F	3		
3G	3		
3H	3		
3J	3		
3K	3		
4B	5		
4F	3		
4H	3		
6B	3		
6D	3		
7B	3		
7D	3		
7E	3		
