# Supplementary material for: Interplay between T3SS effectors, ExoY activation, and cGMP signaling in Pseudomonas aeruginosa infection
Source: Nat Commun. 2025 Dec 2;17:69. doi: 10.1038/s41467-025-66674-z (PMC12770572; doi:10.1038/s41467-025-66674-z)
Supplement: Supplementary file 3 — Description of Additional Supplementary File [file 41467_2025_66674_MOESM3_ESM.pdf]

Description of Additional Supplementary Files

Supplementary Data 1 :

List of strains and plasmids used in this study

Supplementary Data 2:

List of primers used in this study
